# Emerging roles of the HECT E3 ubiquitin ligases in gastric cancer

**DOI:** 10.3389/pore.2023.1610931

**Published:** 2023-02-07

**Authors:** Aiqin Sun, Xianyan Tian, Yifei Chen, Wannian Yang, Qiong Lin

**Affiliations:** ^1^ School of Medicine, Jiangsu University, Zhenjiang, China; ^2^ Department of laboratory Medicine, School of Medicine, Jiangsu University, Zhenjiang, China

**Keywords:** gastric cancer, ubiquitination, tumor suppressor, HECT E3 ubiquitin ligases, oncoprotein

## Abstract

Gastric cancer (GC) is one of the most pernicious gastrointestinal tumors with extraordinarily high incidence and mortality. Ubiquitination modification of cellular signaling proteins has been shown to play important roles in GC tumorigenesis, progression, and prognosis. The E3 ubiquitin ligase is the crucial enzyme in the ubiquitination reaction and determines the specificity of ubiquitination substrates, and thus, the cellular effects. The HECT E3 ligases are the second largest E3 ubiquitin ligase family characterized by containing a HECT domain that has E3 ubiquitin ligase activity. The HECT E3 ubiquitin ligases have been found to engage in GC progression. However, whether HECT E3 ligases function as tumor promoters or tumor suppressors in GC remains controversial. In this review, we will focus on recent discoveries about the role of the HECT E3 ubiquitin ligases, especially members of the NEDD4 and other HECT E3 ligase subfamilies, in GC.

## Introduction

Ubiquitin is an evolutionarily conserved 76-amino acid polypeptide, which serves as a modifier molecule that covalently binds to target proteins for degradation or non-degradative signaling (1). The process attaches ubiquitin to specific substrate proteins, which is called ubiquitination, and is one of the most important protein post-translational modifications. Ubiquitination has been shown to regulate and maintain basic biological functions, including cell cycle, cell differentiation, cell localization, cell survival, apoptosis, signal transduction, and the inflammatory response (2-4). Ubiquitination, including activating, conjugating, and ligating, is a highly orchestrated enzymatic cascade involved in the multistep processes catalyzed by activating enzymes (E1), conjugating enzymes (E2), and ubiquitin ligases (E3) (5). In the human genome, two E1s, 38-40 E2s, and more than 600 E3 ubiquitin ligases have been identified (6,7). E3 ubiquitin ligases are remarkable enzymes, which directly medicate the interaction between ubiquitin and substrates, and mainly determine the substrate specificity and versatility for ubiquitination modification (8,9). In mammalian cells, E3 ligases are usually the prominent focal point of cell regulation, which makes them the most attractive targets for disease treatment and intervention (10,11). HECT E3 ligase activity and substrate specificity can be regulated by a wide variety of mechanisms, such as different post-translational modifications or protein–protein interactions (12). There are several approaches for targeting the HECT E3s, however specific inhibitors of the HECT E3s have still not been exploited.

Gastric cancer (GC) is an extremely aggressive tumor and is estimated to be a major cause of cancer-related death worldwide (13,14). The pathogenetic mechanisms of gastric cancer are complex and heterogeneous, involving environmental factors, gene mutations, copy number variants, and epigenetic changes (15,16). Recently, the HECT E3 ligases have received considerable attention due to their multiple roles in the occurrence and development of GC. Due to the scarcity of studies concentrating on the potential contributions of HERC subfamily E3 ligases to GC, in this review we focus on the roles of NEDD4 subfamily and other HECT E3 ligases in GC development (Table 1).

**TABLE 1:** HECT E3 ligases in human gastric cancer.

E3	Expression	Substrates	Adaptors/Regulators	Pathway	Functions	Role in GC	Reference
NEDD4	upregulation	unknown	Unknown	unknown	regulates proliferation, migration and invasion	dual	(17–20)
NEDD4L	downregulation	unknown	HIF-1α	unknown	unknown	tumor suppressor	(21, 22)
ITCH	downregulation	SMAD7	ASSP2 miR-17 Cir-ITCH	TGF-β pathway Wnt/β-catenin pathway	relates to invasion and TGF-β1-induced EMT	dual	(23–25)
WWP1	upregulation	unknown	miR-584-5p miR-129-5p miR-129-3p	TGF-β pathwayPTEN/AKT pathway	increases tumor growth and decreases apoptosis	oncoprotein	(26–28)
WWP2	upregulation	unknown	unknown	PTEN/PI3K/AKT pathway	promotes proliferation	oncoprotein	(29)
SMURF1	upregulation	MEKK2	miR-424 miR-1254 Kir2.1 STK38	PI3K/AKT pathway MEK1/2–ERK1/2 pathway	participates in metastasis and EMT	dual	(30–33)
HUWE1	upregulation	TGFBR2	unknown	unknown	promotes proliferation, migration, and invasion	oncoprotein	(34)
HACE1	downregulation hypermethylation	cyclin C	unknown	Wnt/β-catenin pathway	suppresses proliferation and migration, enhances apoptosis	tumor suppressor	(35–37)
UBR5	upregulation frameshift mutations	GKN1	unknown	unknown	enhances cell growth, migration, and invasion	oncoprotein	(38–40)
UBE3C	upregulation	P53 AXIN1	RAD18	Wnt/β-catenin pathway P53 pathway	promotes proliferation and inhibits apoptosis	oncoprotein	(41, 42)
HECTD3	upregulation	c-MYC	unknown	unknown	facilitates proliferation and inhibits apoptosis	oncoprotein	(43)

## The HECT E3 ubiquitin ligases

Based on their structures and catalytic mechanism, E3 ubiquitin ligases can be categorized into three groups: HECT (Homologous to E6-associated protein C-terminus) E3s (44-46), RING (really interesting new gene) E3s (47,48), and RBR (RING-between-RING) E3s (49,50). RING E3s, which make up 95% of E3 ligases, act as scaffold proteins, performing a single-step ubiquitin transfer from the ubiquitin-charging E2 enzyme to the substrates (45). Unlike RING E3s, HECT and RBR E3 ligases contain catalytic cysteine residue, forming an intermediate thioester bond with ubiquitin before the transfer of ubiquitin to lysine residue on the substrates (51).

**FIGURE 1:**
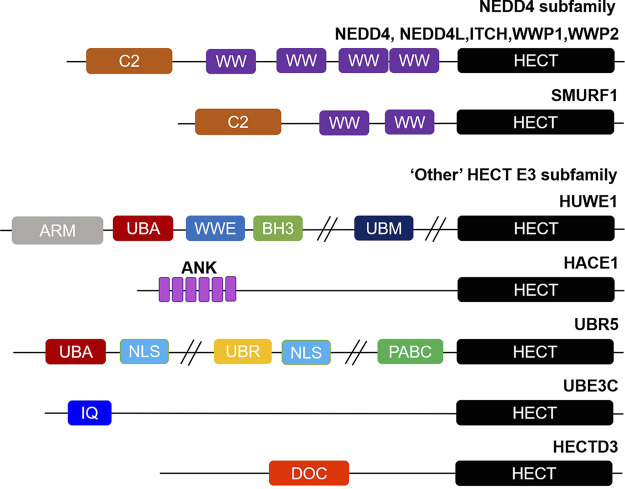
Overview of structural domain organization of HECT E3 family members. HECT E3 ubiquitin ligases are grouped into three subfamilies: NEDD4, HERC, and “Other” HECT E3 subfamily on the basis of their N-terminal structural properties to the HECT domain. The NEDD4 subfamily is characterized by C2 and WW domains, and “Other” HECT E3 subfamily carries a myriad of structural domains in their N-terminal region. The domain abbreviations used are as follows: C2, Calcium-dependent lipid binding domain; WW, WW domain; HECT, homologous to E6AP C-terminus; ARM, armadillo repeat-containing domain; UBA, ubiquitin-associated domain; WWE, WWE domain; BH3, Bcl-2 homology 3 domain; UBM, ubiquitin-binding motif; ANK, Ankyrin repeat-containing domain; NLS, nuclear localization sequence; UBR, ubiquitin-recognin box; PABC//MLLE, polyadenylate-binding protein C-terminal domain; IQ, IQ motif/EF-hand binding site; DOC, APC10/DOC domain.

HECT E3 ligases have a total of 28 members, typically with a conserved ∼40 kDa HECT domain, which can be further divided into three subgroups on the basis of their domain architecture in the N-terminus:NEDD4 subfamily with C2 and WW domains, HERC subfamily with RLD domains, and “Other” HECT E3 subfamily which contains neither C2 and WW domains nor RLD domains (12,52) (Figure 1).

The NEDD4 subfamily is the most famous and has been studied most in-depth of the HECT E3 ligases, consisting of 9 proteins in humans: NEDD4 (NEDD4-1), NEDD4L (NEDD4-2), ITCH (AIP4), WWP1 (AIP5), WWP2 (AIP2), SMURF1, SMURF2, NEDL1 (HECW1) and NEDL2 (HECW2) (53). Structurally, NEDD4 subfamily proteins contain an N-terminal C2 domain, two to four WW domains in the central region, and a C-terminal HECT domain (Figure 1). The C2 domain is defined as a Ca^2+^ and is a phospholipid binder involved in protein-protein interactions, regulating the activity of the HECT domain, as well as membrane binding (54-58). The WW domains have two conserved tryptophan residues, which are thought to be mainly responsible for protein-protein interactions *via* interacting with proline rich PPXY motifs and phosphorylated Ser/Thr residues in substrates (59-61). Whereas the HECT domain exhibits catalytic E3 activity and catalyzes the transfer of ubiquitin to substrates. The HERC subfamily can be divided into large and small HERCs. The large HERCs include HERC1 and HERC2 that contain more than one RLD domain and additional predicted domains. Whereas the four small HERCs, named HERC3∼6, only have a single RLD domain. RLD domains can interact with chromatin through histones H2A and H2B, in addition to functioning as a guanine nucleotide exchange factor (GEF) to regulate the small GTPase Ran (62,63). Finally, the 13 members of other HECT E3s carry a myriad of structural domains in their N-terminal region that are responsible for binding and recruiting substrates (64) (Figure 1). EA6P, HUWE1, UBR5 (EDD), TRIP12 (ULF), and HACE1 are the best studied of other HECT E3 ligases. It is worth emphasizing that with the development of sequencing technology, it may be possible to further subdivide the ‘other’ HECT E3s into subfamilies according to their genetic evolutionary histories in the near future (65,66).

### NEDD4 subfamily

#### NEDD4

Neuronal precursor cell-expressed developmentally downregulated 4 (NEDD4, also known as NEDD4-1) is the ancestral and prototypic member of the NEDD4 family, and has been unveiled as a versatile E3 ligase with an extensive range of target substrates. NEDD4 is thought to be implicated in various human diseases, from cardiovascular disease (Liddle’s syndrome) to cancers (61,67). Interestingly, the relationship of NEDD4 to GC appears to be rather complicated. One study showed that overexpression of NEDD4 was detected in 75% GC tissues (17). Surprisingly, there is no association between NEDD4 levels and invasion, metastasis, or stage. However, another study found that NEDD4 is upregulated in advanced GC tissues and associated with metastasis (18). In accordance with the study, we found that gastric cardia adenocarcinoma patients have a high expression of NEDD4, which was associated with metastasis occurrence and negative prognosis of patients (19). Unexpectedly, Yang et al. observed no upregulation of NEDD4 and no correlation with PTEN expression in GC tumor samples (20). The discrepancy may be due to the differences in gastric tumor types, stages, or sample numbers. Therefore, it is worth exploring the role of NEDD4 in GC further.

#### NEDD4L

Neural precursor cell-expressed developmentally downregulated 4-like (NEDD4L, also known as NEDD4-2) is highly homologous to NEDD4 and is thought to have originated later in evolution, as it has a different substrate repertoire from NEDD4 (68). To date, NEDD4L has been implicated in carcinogenesis and tumor progression by regulation of multiple central pathways, including TGF-β, PI3K-AKT, Wnt, and EGFR signaling pathways (69-72). GC patients were observed to express low levels of NEDD4L with an aggressive clinical course and poor clinical outcomes (21). Additionally, NEDD4L is an independent predictor for GC metastasis and survival. At the same time, the expression of NEDD4L protein in GC tissues is negatively correlated with HIF-1α, suggesting that NEDD4L may, together with HIF-1α, contribute to metastasis of GC (22), but the underlying molecular mechanism remains unclear.

#### ITCH

ITCH, also known as AIP4, was initially identified as an important immune system regulator (73). Emerging studies have revealed a critical connection between ITCH and tumorigenesis, and GC as well. ITCH is reported to ubiquitinate and degrade SMAD7 (74,75). Gen et al. have demonstrated ITCH activity is restricted *via* its interaction with ASSP2 that exerts an inhibitory competitive action on the recruitment of SMAD7, a key gene in TGF-β1-SMAD2/3 signaling, which in turn inactivates TGF-β1-SMAD2/3 signaling to inhibit invasion and TGF-β1-induced EMT in diffuse-type GC (23). However, a recent study found Cir-ITCH may increase ITCH expression by sponging miR-17 (24). Moreover, Cir-ITCH significantly decreases the protein level of β-catenin and p-Dvl2 and two Wnt target genes c-Myc and cyclin D1, thus attenuating the cell proliferation, migration, and invasion in GC (24). Clinically, GC patients expressing low levels of Cir-ITCH and ITCH with metastasis have shorter overall survival times (24,25). Thus, the true role of ITCH in GC is still controversial and further studies will be necessary to identify the downstream substrates and mechanisms.

#### WWP1

WW domain-containing E3 ubiquitin protein ligase 1 (WWP1, also known as AIP5 or TIUL1) is related to NEDD4 family members, contains four WW domains, and is primarily involved in inflammation, neurological disorders, cardiovascular diseases, and malignancies (76) (Figure 1). Elevated expression of WWP1 has been detected in human GC tissues and cells, and has been shown to be associated with poor clinicopathological characteristics and worse survival (26). Besides, downregulation of WWP1 leads to compromised tumor proliferation, as well as enhanced G0/G1-phase arrest and apoptosis by governing the PTEN/AKT signaling pathway (26). Its similar oncogenic activity has also been confirmed in subsequent studies, evidenced by the fact that WWP1 is a direct target of miR-584-5p, miR-129-5p, and miR-129-3p, and these miRNAs inhibit GC tumor growth by targeting WWP1 (27,28). All of these studies indicate that WWP1 is an important tumor promoter and promising target in GC.

#### WWP2

WWP2 (also known as AIP2) functions as an E3 to regulate a myriad of cellular activities (77), which is interestingly emerging to have a role in carcinogenesis. At present, only one study has provided insight into the role of WWP2 in GC. WWP2 was found to be upregulated in GC tissues compared with paired adjacent non-tumor tissues (29). In patients with GC, the upregulated expression of WWP2 contributed to an unfavorable prognosis (29). In addition, depletion of WWP2 increases PTEN protein levels and decreases AKT phosphorylation level, thereby inhibiting the proliferation of GC cells both *in vitro* and *in vivo* (29). However, whether WWP2 regulates PTEN/PI3K/AKT pathway through its E3 ligase activity remains elusive.

#### SMURF1

Like other NEDD4 family members, SMAD-specific E3 ubiquitin protein ligase 1 (SMURF1) interacts with many potential targets and plays a role in different cellular functions including bone homeostasis, embryogenesis, autophagy, and carcinogenesis (78). Deregulated expression of SMURF1 has been reported in several human cancers, and its increased expression is frequently associated with disease progression and prognosis (79). Interestingly, SMURF1 appears to have a dual role in carcinogenesis, the majority of the studies assign SMURF1 a role of oncogenic factor. In GC, highly expressed SMURF1 has been observed in tumor tissues and cell lines, which was associated to malignant phenotypes and decreased survival (30). The pro-tumor property of SMURF1 was attributed to suppress the expression of DAB2IP and subsequently enhances activation of the PI3K/AKT pathway (30). Besides, SMURF1 has been reported to be a target of miR-424, thereby regulating cisplatin-resistant advanced GC (31). Similarly, Jiang et al. have unveiled that miR-1254 could target the 3′-UTR of SMURF1 and inhibit its protein expression to inactivate the PI3K/AKT pathway, thus repressing the proliferation, migration, invasion, and EMT of GC cells (32). Together, the above results point to SMURF1 as an activator of the PI3K/AKT pathway and suggest that it functions as a tumor promoter in GC. Interestingly, SMURF1 mediated pluripotency degradation of MEKK2 was attenuated by the interaction of Kir2.1 with STK38, which resulted in activation of MEK1/2–ERK1/2 signaling also stimulating GC cell invasion and metastasis (33).

### Other HECT E3 ligases

#### HUWE1

The HECT, UBA, and WWE domain-containing E3 ubiquitin protein ligase 1 (HUWE1, also known as Mule, HectH9, ARF-BP1, HSPC272, Ib772, URE-B1, E3Histone, and LASU1) is a large and evolutionarily conserved E3 belonging to the other HECT subfamily (64) (Figure 1). In most cases, HUWE1 could regulate many biological processes, such as DNA damage repair, transcription, cell proliferation, differentiation, autophagy, and apoptosis (80). Available evidence shows that HUWE1 is interestingly emerging to have a role in tumorigenesis, and in GC as well. Very little was known about how HUWE1 regulates GC, until recently, when He et al. reported the role and mechanistic studies of HUWE1 in GC. The results showed that GC patients expressed high levels of HUWE1 and low protein levels of TGFBR2 (34). In addition, overexpression of HUWE1 promoted the proliferative activity, migratory, and invasive potential of GC cells (34). Mechanistically, HUWE1 can degrade the tumor suppressor TGFBR2 through ubiquitination, leading to the malignant progression of GC (34). The extent of HUWE1 contribution to gastric malignancies is still far from being understood.

#### HACE1

In addition to HECT domain, the E3 ligase HACE1 harbors six ankyrin repeats that function to mediate protein-protein interactions (Figure 1). HACE1 has attracted much attention in recent years because of its involvement in the development of human malignancies, in which it acts as a tumor suppressor (81). Chen et al. found that HACE1 was significantly reduced in GC tissues and cell lines, and it was closely correlated with tumor pathological differentiation (35). Accordingly, lower HACE1 expression is associated with better overall survival rate in TNM stages I-IIIa GC patients (35). In addition, overexpression of HACE1 suppressed the ability of proliferation and migration of GC cells, and enhanced cell apoptosis (35). Mechanistically, HACE1 can downregulate the expression of β-catenin, which in turn inhibits the activity of the Wnt signal to impede the malignant progression of GC (35). Furthermore, the authors demonstrated HACE1 executes its regulation of the Wnt/β-catenin signaling through its E3 ligase activity (35). However, the specific substrates in the Wnt/β-catenin signaling of HACE1 in GC are still largely unknown. Besides, HACE1 has been reported to cause ubiquitination of cyclin C in the non-proteolytic K11 linkage form, which led to the cisplatin-induced nuclear–mitochondrial translocation of cyclin C and finally promoted apoptosis of GC cells (36). The investigation of Sakata M et al. showed that hypermethylation of the HACE1 gene is associated with reduced expression of HACE1 (37), which points out that demethylation of HACE1 may be a new strategy for the treatment of GC.

#### UBR5

The other HECT E3 ligase UBR5 (also known as EDD1, EDD, DD5, HYD, or KIAA0896), consists of a ubiquitin-binding UBA domain, a zinc finger-like UBR domain involved in N-rule substrate recognition, two nuclear localization sequences, a PABC/MLLE domain for protein–protein interaction, and a C-terminal HECT (82) (Figure 1). UBR5 has been related to the regulation of DNA damage response, translation, metabolism, transcription, and apoptosis (83). Recent evidence has unveiled a critical role of UBR5 (38)in the progression of multiple cancer types, including GC. UBR5 was mutated in 27.8% of GC, and GC patients with high UBR5 expression had a poor prognosis (39,40). Moreover, Knockdown of UBR5 in GC cells was shown to suppress cell growth, migration and invasion, which is mainly due to the inhibition of protein stability of tumor suppressor GKN1 through ubiquitination (84).

#### UBE3C

UBE3C, also known as RAUL, contains an N-terminal IQ motif and a C-terminal HECT domain (Figure 1), and is able to assemble K48 and less preponderant K29 and K11 linkages (85). A study identified the N-terminal region just preceding the HECT domain is crucial for the stabilization and enzymatic activity of UBE3C (86). Accumulating evidence indicated that UBE3C is implicated in several tumors, including glioma, melanoma, renal cell carcinoma, hepatocellular carcinoma, breast cancer, lung cancer, and gastric cancer (41,42,87-91). And the role of UBE3C in gastric cancer has only recently begun to be explored. Zhao et al. discovered that LINC00355, as a tumor promoter, promotes the malignant progression of GC by enhancing the association of E3 ubiquitin ligases UBE3C and RAD18 with P53, facilitating its ubiquitination and degradation (43). Furthermore, another study identified that the protein and mRNA levels of UBE3C in GC were both heightened and inversely correlated with patient outcomes. UBE3C could interact directly with and downregulate AXIN1, thus activating β-catenin signaling and promoting the development and progression of GC (42).

#### HECTD3

Homologous to the E6-associated protein carboxyl terminus domain containing 3 (HECTD3) is an under-investigated other HECT E3 ligase and characterized by an N-terminal DOC domain (Figure 1). Recently, Zhang et al. discovered the abnormal activation of HECTD3 in GC cells, which was correlated with poor outcomes of patients (92). Also, HECTD3 could facilitate the malignant proliferation and tumorigenesis of GC cells, and inhibit the cell apoptosis, suggesting that it serves as a tumor promoter in GC. Mechanistically, HECTD can stabilize expression of c-MYC by interacting with c-MYC and mediating K29 linked polyubiquitination of c-MYC (92).

## Conclusion and perspective

**FIGURE 2:**
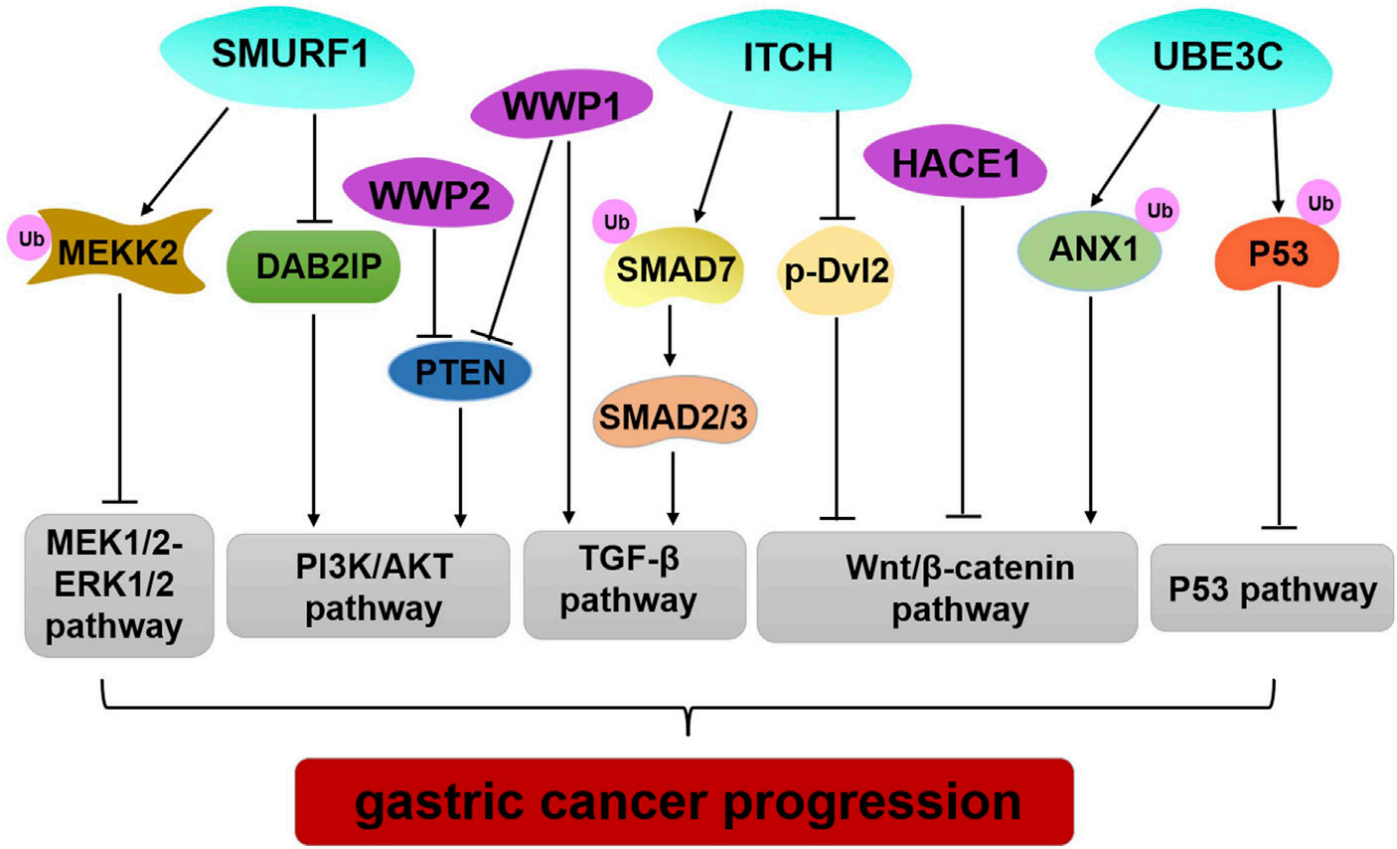
Regulation of important signaling pathways by HECT E3 ligases in GC. SMURF1 mediated pluripotency degradation of MEKK2, which resulted in inactivation of MEK1/2–ERK1/2 signaling. SMURF1, WWP1, and WWP2 have similar functions by regulating PIAK/AKT signaling pathway. Limiting ITCH activity causes accumulation of SMAD7 and blocks TGF- β1 signal transduction to inhibit GC progression. ITCH also exerts tumor-suppressive functions by down-regulating the protein level of β-catenin and p-Dvl2. HACE1 executes its regulation of the Wnt/β-catenin signaling pathway through its E3 ligase activity. UBE3C promotes ubiquitination and degradation ANX1 to activate the WNT/β-catenin signaling in GC development. UBE3C directly binds to p53, and it mediates p53 degradation to inhibit the p53 pathway in GC.

The data reviewed here illustrate genetic alteration, abnormal expression, or dysfunction of HECT E3 ligases that may contribute to GC occurrence and progression (Table 1; Figure 2). However, several HECT E3 ligases have been reported to play a dual role in GC (Table 1). The concrete mechanisms have not been fully explored. Currently, E3 ubiquitin ligases become important therapeutic targets for cancer treatment (10,93). Many inhibitors of the RING family E3 ligases have been developed for cancer therapy, while only limited efforts have been made regarding the HECT E3 ligases. With the better understanding of the mechanism underlying regulation of the HECT E3 ubiquitin ligases, and advances in research technology, such as high-throughput screenings and PROTAC (proteolysis targeting chimera) technology, we will be able to develop more drugs that target the HECT E3 ligases for GC therapy.
